# Syncope in a Patient with Right Ventricular Compression from Severe Pectus Excavatum: A Case Report

**DOI:** 10.5811/cpcem.50611

**Published:** 2026-01-14

**Authors:** Matthew J. Christensen, Jennifer Foti

**Affiliations:** Naval Medical Center San Diego, Department of Emergency Medicine, San Diego, California

**Keywords:** syncope, pectus excavatum, right ventricle compression, case report

## Abstract

**Introduction:**

Pectus excavatum (PEX) is the most common congenital chest wall deformity, characterized by posterior depression of the sternum and lower costal margin. While often asymptomatic, severe PEX can lead to compression of the heart and great vessels, potentially causing right ventricular dysfunction, syncope, and other cardiovascular symptoms. Syncope due to right ventricle compression in PEX is rare but can significantly impact quality of life and may require surgical intervention.

**Case Report:**

An 18-year-old female presented to the emergency department after an unwitnessed syncopal episode. The patient reported feeling lightheaded while showering, followed by collapse and brief loss of consciousness. Diagnostic testing revealed normal neurological and metabolic parameters including point-of-care glucose, electrocardiogram, serum troponin, electrolytes, and head computed tomography. Chest imaging showed severe PEX with concerns of right ventricular compression. Transthoracic echocardiography demonstrated normal cardiac function, and exercise stress testing showed no ischemic changes. Additional laboratory studies revealed iron deficiency anemia.

**Conclusion:**

This case underscores the potential for PEX to cause distorted cardiac morphology, including right ventricular compression, which can lead to syncope in severe cases. The absence of cardiac ischemia, arrhythmias, or metabolic derangement suggests postural changes compounded by undiagnosed anemia and underlying PEX as the most likely cause of this patient’s syncope. Given the patient’s symptoms and anatomical findings, referral for surgical evaluation was made to discuss definitive management options. This case highlights the importance of considering structural chest wall abnormalities in the differential diagnosis of syncopal events, particularly when standard causes are excluded.

## INTRODUCTION

Pectus excavatum (PEX) is a congenital chest wall deformity characterized by posterior depression of the sternum and lower costal margin.^1^ This condition is the most common structural anomaly of the anterior chest, occurring in approximately 1 in 300–400 live births.^1^ Females with PEX typically experience more severe chest wall deformity than males, although this does not lead to worse exercise intolerance or overall cardiopulmonary function.^2^ In severe cases, the chest wall deformity can lead to physical compression of mediastinal structures including the heart and great vessels, which has been implicated in cardiovascular pathology including ventricular arrhythmias,^3^,^4^ palpitations,^5^ right ventricle dysfunction,^6^,^7^,^8^ and sudden cardiac arrest.^9^,^10^

While PEX does not cause left ventricle dysfunction,^11^ the most notable hemodynamic impact of the resultant distorted right ventricle morphology is the potential for clinically significant ventricular compression, which can predispose to syncope or near-syncopal events.^12^,^13^ In severe cases of PEX, the posterior displacement of the sternum can compress the right ventricle and inferior vena cava, which reduces venous return and impairs right ventricular filling.^14^,^15^ This functionally decreases cardiac output, particularly during physical exertion or in postural changes, when the heart’s ability to adapt to changes in intrathoracic pressure becomes strained. This impaired cardiac output may cause a drop in cerebral perfusion, leading to syncope or near-syncopal episodes. Although syncope in PEX patients is less common than other symptoms such as exercise intolerance, chest pain, or dysrhythmias, it is an important clinical manifestation that can significantly impact quality of life and may indicate a need for surgical intervention.

## CASE REPORT

An 18-year-old female with no prior medical history was brought by ambulance to the emergency department after an unwitnessed syncopal event that occurred at her home. The patient reported that she was in the shower, felt lightheaded, stepped out of the shower, and then collapsed into the bathroom counter. Her roommate heard the noise and discovered her on the floor, with loss of consciousness lasting approximately one minute. No convulsive activity or post-ictal state was witnessed. Physical exam revealed no focal neurologic deficits or sequelae of head trauma, although there was mild tenderness to palpation of the lateral left chest wall. Point-of-care blood glucose was normal. Electrocardiogram showed no dysrhythmias and no ischemic changes. Serum troponin-I level was undetectable.

Complete metabolic panel demonstrated normal kidney function and normal liver function, and was without electrolyte derangement. Complete blood count revealed a stable microcytic anemia (hemoglobin 8.9 grams per deciliter (g/dL) (reference range: 12.0–16.0 g/dL) and hematocrit 26.7% (36%–46%) (compared to hemoglobin 8.7 g/dL and hematocrit 26% measured seven months prior) without leukocytosis, leukopenia, or thrombocytopenia. Urine pregnancy testing was negative. Non-contrast computed tomography (CT) of the head was obtained due to the traumatic mechanism and reported headache, which showed no intracranial pathology.

**Image 1:**
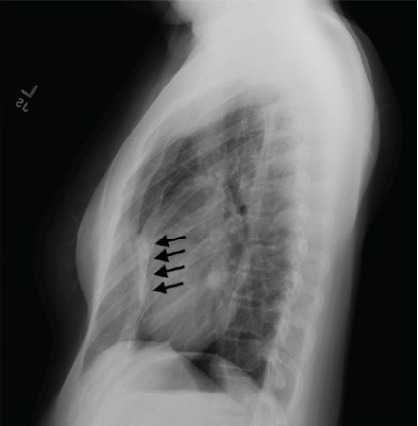
Chest radiograph (lateral view) demonstrating right ventricular compression from severe pectus excavatum (black arrows).

**Image 2:**
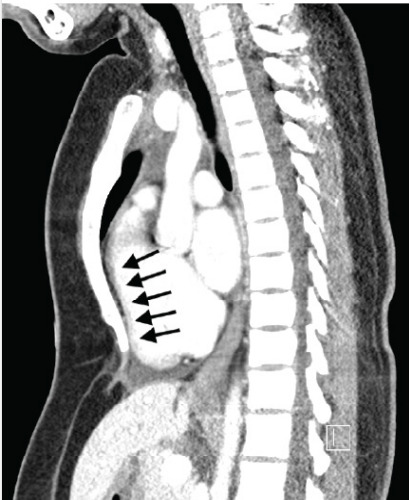
Contrast-enhanced chest computed tomography (sagittal plane) demonstrating right ventricular compression from severe pectus excavatum (black arrows).

**Image 3:**
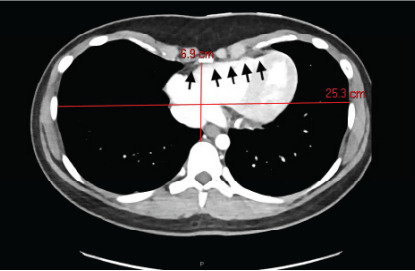
Contrast-enhanced chest computed tomography (axial plane) demonstrating right ventricular compression from severe pectus excavatum (black arrows). Thoracic cavity measurements are included in red to highlight the abnormal anterior-posterior diameter of this patient’s chest wall.

A two-view chest radiograph was obtained due to the reported left-sided rib pain with tenderness on exam, which showed no apparent rib fractures, pulmonary contusion, or pneumothorax, but did reveal severe PEX with concern for right ventricular compression (Image 1). Contrast-enhanced CT of the chest was ordered for further characterization, which demonstrated significantly reduced anterior-posterior diameter of the chest and right sternal torsion with resultant compression of the right ventricle (Images 2 and 3). The patient was admitted to the cardiology service for additional diagnostic testing, telemetry monitoring, and risk stratification.

*CPC-EM Capsule*

What do we already know about this clinical entity?

*Pectus excavatum is the most common congenital chest wall deformity, and has been implicated in cardiovascular pathology including ventricular arrhythmias and cardiac arrest.*

What makes this presentation of disease reportable?

*This is the first published case report of syncope caused by right ventricular compression from severe pectus excavatum.*

What is the major learning point?

*Consider atypical etiologies such as cardiac compression from structural chest wall deformity in patients with syncope when standard causes have been excluded.*

How might this improve emergency medicine practice?

*Broadening our differential diagnoses for common emergency department presentations will improve the care we provide to our patients.*

Transthoracic echocardiogram revealed left ventricular ejection fraction of 60% with normal right ventricle and left ventricle function and no evidence of right ventricular collapse, right atrial dilation, or elevated pulmonary artery pressure. Exercise stress testing showed no ischemic changes during exercise or recovery. Continuous telemetry revealed no paroxysmal dysrhythmias during her hospital course. Iron studies revealed iron deficiency as the likely cause of the identified microcytic anemia. The patient was ultimately discharged after a three-day hospitalization with outpatient referral to cardiothoracic surgery to discuss surgical and nonsurgical management options.

## DISCUSSION

Several clinical learning points are demonstrated throughout this case. It highlights the importance of maintaining a broad differential diagnosis in syncope, the impact of PEX on cardiac morphology, and the potential compounding effect of an underlying microcytic anemia in PEX that may predispose to syncopal events even in the absence of overt cardiac dysfunction.

Syncope, defined as a transient loss of consciousness due to insufficient cerebral perfusion,^1^ requires a comprehensive workup to identify the underlying cause. Although syncope or near-syncopal episodes in young and otherwise healthy patients are often the result of benign vasovagal or orthostatic etiologies, this case emphasizes the importance of considering a broad differential diagnosis even in the healthy population. This patient’s normal physical exam, normal point-of-care glucose, unremarkable electrocardiogram, normal serum troponin-I, and unremarkable head CT all lowered the likelihood of classic emergent causes of syncope such as hypoglycemia, dysrhythmias, cardiac ischemia, or intracranial hemorrhage. Syncopal events may also be provoked by anemia or impaired cardiac function. This patient’s radiographic evidence of severe PEX with right ventricle compression along with laboratory evidence of chronic microcytic anemia likely had compounding effects that contributed to her syncope.

Severe PEX may cause physical compression of critical cardiac structures, particularly the right ventricle. 6,7,8 This case demonstrates how imaging studies such as a chest radiograph or contrast-enhanced chest CT can reveal underlying structural abnormalities in the appropriate clinical scenario, which might otherwise be overlooked. Resultant right ventricle compression may impede normal blood flow and cause syncope, especially during activities that decrease venous return. This patient’s syncopal event occurred in the shower, potentially due to postural changes or increased intrathoracic pressure, and was likely compounded by the underlying chronic microcytic anemia.

Another key element in this case is the identification of microcytic anemia. The patient’s stable hemoglobin (8.9 g/dL) and hematocrit (26.7%) compared to values from seven months prior (hemoglobin 8.7 g/dL and hematocrit 26%), as well as the new diagnosis of iron deficiency anemia based on inpatient iron studies, suggest that a previously undiagnosed chronic anemia likely contributed to her syncope. Acute blood-loss anemia is less likely in this patient without reported menorrhagia and without melena or hematochezia to suggest occult gastrointestinal bleed. Anemia can impair oxygen delivery, especially in the context of structural cardiac abnormalities such as PEX, which further reduces cardiac output and may exacerbate symptoms. This case underscores the importance of considering anemia as a contributing factor in patients with unexplained syncope, and the value of routine laboratory tests such as a complete blood count and iron studies when indicated.

The patient’s management involved careful risk stratification including cardiac imaging, echocardiography, and exercise stress testing, which revealed no major functional abnormalities or ischemia. Given the absence of acute cardiac compromise, she was discharged with outpatient follow-up for cardiothoracic surgical consultation, which may alleviate the structural compression and mitigate the risk of future syncopal episodes. This case demonstrates the importance of multidisciplinary management, as the etiology of her iron deficiency anemia will require additional outpatient workup and the decision between surgical and nonsurgical management of PEX will require input from both cardiology and cardiothoracic surgery specialists.

## CONCLUSION

This case demonstrates the importance of a broad differential diagnosis and thorough workup in patients presenting for syncope. Chest wall deformities such as pectus excavatum may lead to cardiovascular compromise, and a comprehensive approach that considers both structural and non-structural causes of syncope is essential.
